# Exploring the roles of genes associated with leukocyte transendothelial migration in liver cirrhosis development

**DOI:** 10.1371/journal.pone.0350886

**Published:** 2026-06-03

**Authors:** Lei Feng, Jiao He, YanLing Mu, Jie Liu, Yong Yao

**Affiliations:** 1 The Division of Gastroenterology and Hepatology, Suining Central Hospital,‌‌ Suining, Sichuan, China; 2 The Pathology Department, Suining Central Hospital, Suining,‌‌ Sichuan, China; Guangdong Nephrotic Drug Engineering Technology Research Center, Institute of Consun Co. for Chinese Medicine in Kidney Diseases, CHINA

## Abstract

**Background:**

Individuals with liver cirrhosis (LC) have an exceptionally high mortality rate. We aim to discover new biomarkers or new therapeutic targets for LC early diagnosis and treatment.

**Methods:**

The human LC microarray datasets were obtained from the NCBI-GEO database. Firstly, differentially expressed analysis and WGCNA were performed to detect LC key genes. Protein-protein interaction (PPI) hubs revealed new regulatory mechanisms for LC-related genes. Functional enrichment and immune infiltration analyses were employed to reveal underlying mechanisms of LC progression. Then, key genes were further screened for constructing diagnostic nomogram for LC and predicting the prognosis of LC. CB-Dock-2 was employed to explore potential therapeutic agents for LC based on the identified key genes.Finally, immunohistochemistry was performed to detect the expression levels of the key genes in liver tissues from patients with liver cirrhosis and healthy controls.

**Results:**

The integrated LC dataset identified 749 LC key genes by intersecting WGCNA and differential expression analyses. PPI analysis identified 15 genes as key regulators in LC development, predominantly enriched in inflammatory and immune regulatory pathways. We conducted an in-depth analysis of two leukocyte transendothelial migration (LTM)-associated genes (MMP2 and ITGB2) among these 15 hub genes, revealing their involvement in the construction of the immune microenvironment in LC. A nomogram based on MMP2 and ITGB2 had favorable diagnostic performance for LC, and ITGB2 showed potential in predicting LC prognosis. Through molecular docking approaches, this study further identified several antioxidant and anti-inflammatory agents with therapeutic potential for LC. Immunohistochemical staining revealed significantly elevated protein levels of MMP2 and ITGB2 in liver tissues from cirrhotic patients compared to healthy controls.

**Conclusions:**

LTM-associated genes, namely MMP2 and ITGB2, are associated with LC and may represent candidate biomarkers or potential targets requiring further experimental validation.

## 1. Introduction

Liver cirrhosis (LC), a top cause of end-stage liver disease, is considered the precursor to hepatocellular carcinoma, affecting about 110 million people worldwide [1,2]. Generally, the various stages of the LC process include the asymptomatic, compensated stage, and the subsequent decompensated stage (i.e., the appearance of typical clinical symptoms associated with liver function abnormalities and portal hypertension). Although compensated cirrhosis patients may remain asymptomatic for many years, about 3%−4% of compensated LC patients will progress to decompensated LC per year [3]. LC, the late stage of liver fibrosis, is pathologically characterized by the excessive hepatic deposition of fibrous tissue as a result of chronic liver inflammation caused by multiple etiological factors including viral hepatitis, metabolic disorders, alcoholic steatohepatitis, non-alcoholic steatohepatitis, and autoimmune diseases [4,5]. Clinical studies suggest that LC is an irreversible, progressive, and ultimately life-threatening liver disease with a poor prognosis [6]. Currently, treatments focused on etiologic factors and potential emerging vital complications are the mainstay of managing most LC patients [7]. However, besides liver transplantation, there is no cure for LC [8]. Thus, more mechanistic research is needed to explore the innovative therapeutic targets for treating LC.

Leukocyte transendothelial migration (LTM) is defined as leukocytes adhering to and extravasating through the vascular endothelium to enter the surrounding tissue, playing an important role in innate immunity and inflammation [9]. Previous studies have reported that LTM is related to the occurrence and development of multiple diseases, including lung, kidney, and liver disease [10–12]. For example, cisplatin-induced kidney injury occurs depending on the recruitment of leukocytes into the kidney [13]. Notably, LTM is also considered an important mechanism involved in the pathological process of liver fibrosis. Several studies have shown that T regulatory cells (Tregs), a subset of leukocytes, can inhibit both adaptive and innate immunity in the hepatic microenvironment, and their migration is associated with the crosstalk between Tregs and other immune cells, involving in the induction of liver fibrosis [14–16]. Thus, the regulation of LTM may serve as a potential therapeutic target for LC. Until recently, however, little research has been done to investigate the relationship between the genes regulating LTM and LC. So, screening out the key genes regulating LTM in LC is necessary.

The present study downloaded the microarray datasets for normal liver and LC tissues from a public database. We then identified the genes highly upregulated in LC tissues and screened for LTM-related genes, using bioinformatics methods. Finally, wet lab experiments were performed to validate our results preliminarily. Our findings may provide novel directions for future treatment of LC, and contribute to improving patient outcomes.

## 2. Materials and methods

### 2.1. Microarray dataset acquisition and process

The raw gene expression datasets for LC and healthy control groups, including GSE14323, GSE25097, GSE77627, GSE139602, and GSE15654, were obtained from the Gene Expression Omnibus (GEO) database (https://www.ncbi.nlm.nih.gov/geo/). Detailed information on the datasets is provided in Table 1. We next integrated two datasets (GSE 14323 and GSE 25097) using the ComBat method in the SVA R package and obtained the integrated LC expression dataset. GSE139602, GSE77627, and GSE15654 microarray datasets were used as external validation sets.

**Table 1:** Descriptive statistics of the GEO Datasets.

GEO accession	Platform	Origin	Sample		LC etiology	Species
			Control	LC		
GSE14323	GPL571	Liver tissue	19	41	Hepatitis C virus	Homo sapiens
GSE25097	GPL10687	Liver tissue	6	40	–	Homo sapiens
GSE139602	GPL13667	Liver tissue	6	28	Alcohol, Hepatitis C virus, NASH, Mixed	Homo sapiens
GSE77627	GPL14951	Liver tissue	14	22	–	Homo sapiens
GSE15654	GPL8432	Liver tissue	–	216	Hepatitis C virus	Homo sapiens

### 2.2. Weighted Gene Co-Expression Network Analysis (WGCNA) and identification of key module genes

WGCNA, a systematic biological approach, was carried out using R package WGCNA to identify the associations of sets of genes and external, biological traits, and find modules of genes highly correlated with phenotype. Before WGCNA, we excluded the outlier samples using the Hclust function available from the“Stats” package of the R programming language.To ensure biological relevance and reduce technical noise, we pre-filtered the gene expression matrix to include only highly variable genes. The selection criterion was based on the relative dispersion of gene expression. We retained genes where the ratio of the standard deviation to the mean expression value exceeded 50%. This step effectively filters out genes with stable/low expression, focusing the network analysis on dynamically regulated transcripts. Samples and genes were flagged as outliers if they exhibited an excessive number of missing entries. Specifically, we applied a stringent cutoff, setting the minFraction parameter to 0.8. The appropriate soft-thresholding power β was established based on the function of “pickSoftThreshold” in the WGCNA package in the statistical R programming language. We then constructed a hierarchical clustering dendrogram and created gene modules according to the similarity of gene expression patterns. Among the identified modules, we merged the highly correlated modules using the mergeCloseModules function in the WGCNA package. The module eigengene (ME) represented the expression profiles of module genes, which was calculated by the MEs function of the WGCNA package. To evaluate the association between co-expression modules and LC, MEs were correlated with the trait, which was coded as a binary variable (0 = Control, 1 = LC). Genes within modules exhibiting high correlation coefficients with this binary LC trait were subsequently selected for further analysis.

### 2.3. Identification of differentially expressed genes (DEGs)

DEGs between LC and healthy controls in the integrated LC expression dataset were identified by the R package “limma”. We defined DEGs by setting an adjusted p-value threshold of <0.05 and a fold-change threshold of at least 2. We used the ggplot2 package and pheatmap package in R statistical software to visualize the expression patterns of DEGs.

### 2.4. Protein-protein interaction (PPI) networks construction and key genes identification

We imported DEGs into the Search Tool for the Retrieval of Interacting Genes (STRING, https://string-db.org/) to construct a PPI network, helping to determine their direct interacting partners and classify the functional gene modules by clustering of PPI network. STRING database provides protein interaction from multiple lines of evidence, including experiments, literature co-occurrence, database, text-mining, gene neighborhood, gene co-expression, and gene fusion. We used four algorithms (MNC, EPC, Degree, and Closeness) in the cytoHubba plugin of Cytoscape software to calculate the top 20 hub genes. Taking the intersection of predicted hub genes, the common genes were determined for further analysis.

### 2.5. Kyoto Encyclopedia of Genes and Genomes (KEGG) and Gene Ontology (GO) enrichment analyses

GO and KEGG pathway analysis of the common genes was performed using the “clusterProfiler” package in the R statistical programming language. The results were presented in the form of corresponding bubble charts.

### 2.6. Immune infiltration analysis

We used the “CIBERSORT” package of R to estimate the proportion of infiltrating immune cell subsets in the study samples. The bar plots depicting the proportion and abundance of the immune infiltration for each sample were generated using the ggplot2 R package. Mann-Whitney test was used to compare the differences in the infiltration proportion of the 22 immune cell subpopulations between LC and healthy control samples, which was presented with a Stacked histogram produced using the ggplot2 package in R. The correlation of 22 types of immune infiltrating cells was displayed using the corrplot R package. Finally, correlation analysis between the immune cell infiltration and the expression of LTM-related genes was performed using Spearman correlations.

### 2.7. Development and validation of a diagnostic nomogram and the prognostic and predictive value of LTM-related genes

Based on the two hub LTM-related genes, a diagnostic nomogram was constructed with the RMS package in R software. Receiving operating characteristics (ROC) curves were created and area under ROC curve (AUC) values were calculated to assess the potential diagnostic value of each hub gene and the nomogram for detecting LC. The decision curve analysis (DCA) and calibration curves were performed to evaluate the nomogram model performance. The Kaplan-Meier survival curves were generated and p values were calculated by Log-Rank test in R programming language using survival R package.

### 2.8. Molecular docking-based drug screening

We used existing drug libraries to screen small-molecule compounds that inhibit the proteins encoded by the hub LTM-related genes in LC patients. First, the catalog of small-molecule compounds that interacting with hub genes was obtained from the Comparative Toxicogenomics Database (CTD, http://ctdbase.org/) and these compound structures were downloaded from PubChem database (https://pubchem.ncbi.nlm.nih.gov). Next, the conformational interaction between small-molecule compounds and selected proteins was performed by using the Cavity detection-guided Blind Docking-2 (CB-Dock-2) tool (http://clab.labshare.cn/cb-dock/php/index.php). CB-Dock-2 can produce multiple protein-small molecule complex models and corresponding model parameters, including the Vina score and the amino acids involved [17]. The criteria for optimal model selection were based on the number involved amino acids greater than 20 and the Vina score (binding affinity) less than −7.0 kJ/mol.

### 2.9. Immunohistochemistry (IHC)

Ethical approval was provided by the ethics committee of Suining central hospital and all subjects gave written informed consent. All tissues were embedded in paraffin and cut into 4µm sections. For immunohistochemical analysis, paraffin sections were dewaxed in xylene and rehydrated through ethanol solutions before antigen retrieval. Heat-mediated antigen retrieval was then carried out by microwaving in boiling citrate buffer (pH 6.0). Following antigen blocking, tissue sections were incubated with rabbit polyclonal anti-MMP2 (1:200, Proteintech Group, China) or rabbit polyclonal anti-ITGB2 (1:200, Affinity Biosciences, China) overnight at 4°C. SP immunohistochemistry kit was purchased from Dako (Copenhagen, Denmark). All tissue sections were histologically diagnosed by two experienced pathologists independently. Immunohistochemical semi-quantification was performed using ImageJ-Fiji software with the IHC Toolbox plugin. Five images from each group were analyzed to measure the average optical density.

### 2.10. Statistical analysis

All visualization and analyses were conducted with R software (version.4.0.5). Two group comparisons were performed by unpaired or paired t-test. P < 0.05 was considered statistically significant.

## 3. Results

### 3.1. Data acquisition and processing

**Fig 1:**
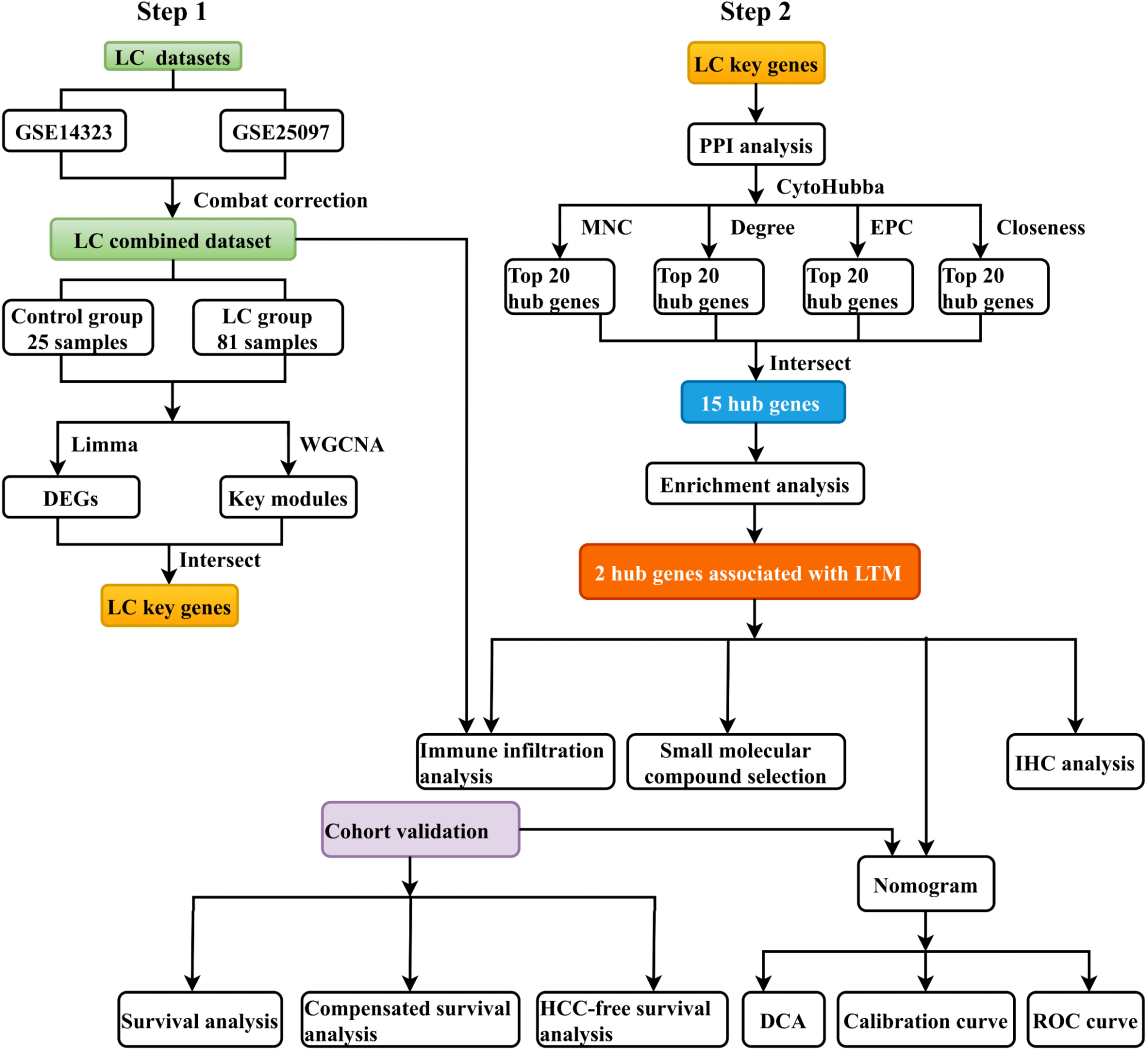
Flow chart of the study.

The bioinformatics analysis strategy is carried out as shown in Fig 1. Two raw datasets(GSE 14323 and GSE 25097) of LC and healthy control group, were retrieved from the GEO database and combined after the batch effect was eliminated. After batch effect rectification, a unified LC dataset was formed and standardized. It encompassed 81 LC tissue specimens in the LC cohort and 25 healthy liver tissue samples as the controls.

Furthermore, we obtained three datasets (GSE139602, GSE77627, and GSE15654) from the GEO database as external validation datasets. The GSE77627 (22 cirrhotic and 14 healthy samples) and GSE139602 (28 cirrhotic and 6 healthy samples) datasets were utilized as validation cohorts to assess diagnostic efficacy of the key genes for LC; meanwhile, the GSE15654 dataset (216 LC tissue specimens) was employed to evaluate the prognostic ability of the key genes for LC.

### 3.2. WGCNA network construction and key modules identification

**Fig 2:**
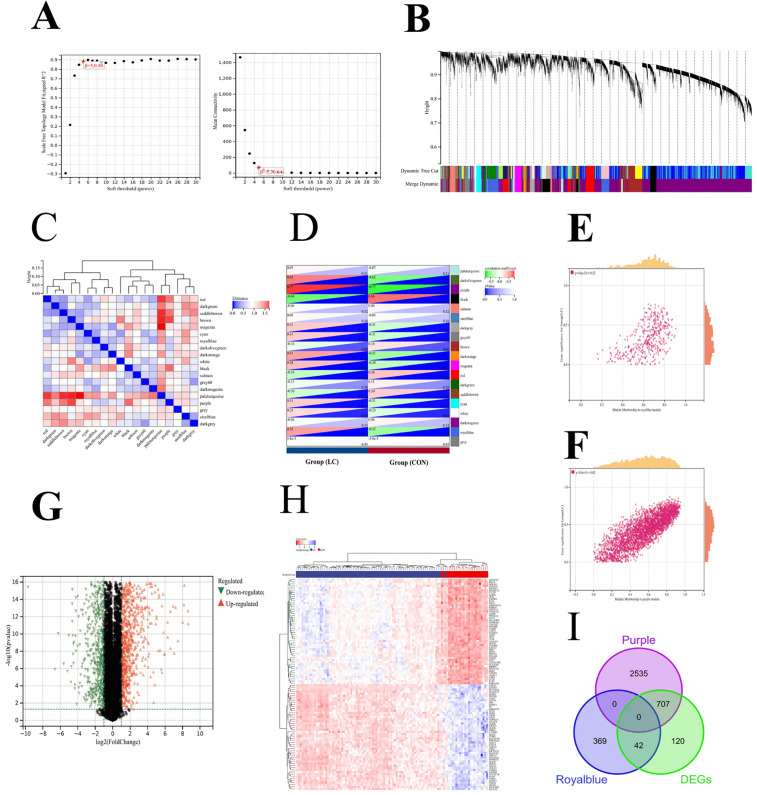
Screening of key module genes in the integrated liver cirrhosis(LC) dataset via WGCNA and identification of LC key genes through the intersection of key module genes and differentially expressed genes(DEGs). **(A)** The scale-free topology model was utilized to identify the best β value, and β = 5 was chosen as the soft threshold based on the average connectivity and scale independence. **(B)** The cluster dendrogram of co-expression genes in LC. **(C)** The cluster dendrogram presenting module eigengenes. **(D)** Module-trait relationships in LC. Each cell contains the corresponding correlation and p-value. **(E)** The correlation plot between the royalblue module membership and the gene significance of genes in the royalblue module. **(F)** The correlation plot between the purple module membership and the gene significance of genes in the purple module. **(G)** Volcano plot of DEGs between LC and control samples(CON). **(H)** Heat map showing the top 50 up and downregulated genes. **(I)** A total of 749 key genes in LC were identified by taking the intersection between key modules genes and DEGs‌‌ via the venn diagram.

WGCNA analyses were conducted to find network modules of highly correlated LC in the unified LC dataset. The soft-thresholding power of 5 was determined by a comprehensive assessment of network topology properties. We aimed to attain a high scale-free topology fit index (R^2^) while maintaining biologically meaningful connectivity. As shown in our power analysis (Fig 2A), a power of 5 was sufficient to achieve an adequate scale-free fit (R² > 0.8) and resulted in a reasonable mean connectivity decay, striking an optimal balance between network connectivity and scale-free topology compliance. Following unsigned network construction, we employed dynamic tree-cutting algorithms to identify modules of co-expressed genes within the unified LC dataset. To ensure robust statistical power for downstream analysis, a cutoff of 30 genes was set as the minimum size for module detection (Fig 2B). Subsequently, to reduce redundancy, modules exhibiting high similarity were merged using a height cutoff of 0.25.The eigenvectors for each WGCNA module were calculated and clustered (Fig 2C). A total of 19 co-expression modules were clustered and the purple module showed the strongest positive correlation with LC (Cor = 0.77, P = 4.8e-22) (Fig 2D). Not only that, the positive correlations were observed between Module Membership (MM) and Gene Significance (GS) in the purple (Cor = 0.82) (Fig 2F) and royalblue (Cor = 0.52) (Fig 2E) modules. Given the strong positive correlations observed with the LC trait and the sufficient number of genes within these modules, the purple and royalblue modules were selected for further analysis.

### 3.3. Identification of differentially expressed genes in LC

Differential analysis between combined LC and control samples revealed 1472 differentially expressed genes (DEGs), containing 869 up-regulated and 603 down-regulated genes. A Volcano plot was used to show DEGs between LC and control samples (Fig 2G). Expressions of the 50 most significantly up- and down-regulated DEGs were displayed in a heat map (Fig 2H). To identify key genes driving the pathogenesis and progression of LC, we focused on genes exhibiting high expression in the disease state. We then took the intersection of up-regulated DEGs with the genes from the purple and royalblue modules identified by WGCNA. This analysis yielded a total of 749 overlapping genes (Fig 2I), which were selected for subsequent analysis.

### 3.4. Construction of a protein-protein interaction (PPI) network and functional enrichment of the Hub genes in LC

**Fig 3:**
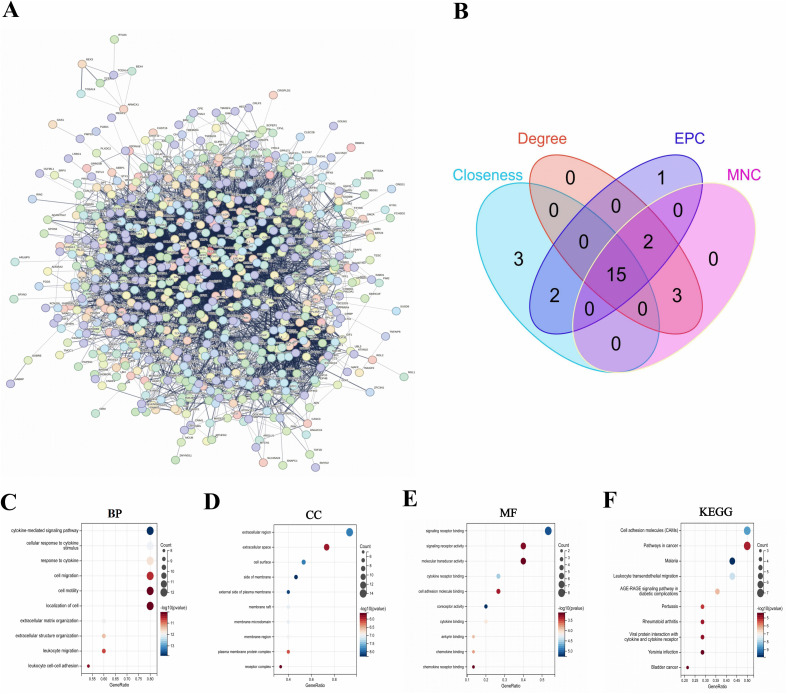
Protein-protein interaction(PPI) network and characterization of the hub genes in liver cirrhosis(LC). **(A)** PPI network of 749 key genes. **(B)** Various algorithms (MNC, Closeness, Degree, and EPC) obtain a Venn diagram of a common gene. The enriched gene ontology terms of the 15 hub genes: **(C)** Biological processes(BP), **(D)** Cellular components (CC), **(E)** Molecular function (MF), and **(F)** Kyoto Encyclopedia of Genes and Genomes (KEGG).

To identify the potential hub genes and underlying mechanism in LC progression, the 749 overlapping genes was analyzed using the STRING database based on a medium confidence score of > 0.4 (Fig 3A). Subsequently, network analysis was conducted using Cytoscape software and the cytoHubba plugin of Cytoscape was used to identify the hub genes in the PPI network. As a result, we found the top 15 overlapping genes from four topological ranking algorithms, including Maximum Neighborhood Component (MNC), Closeness, Degree, and Edge percolated component (EPC) (Fig 3B). Therefore, we considered the top 15 overlapping genes to be the hub genes, including IL-6, PTPRC, CD44, CDH1, CCL2, PECAM1, HIF1A, CXCR4, CD8A, ITGB1, MMP2, CXCL8, VCAM1, ITGB2, and PXDN. To better understand the regulatory mechanisms of the hub genes, we imported the 15 hub genes into DAVID online database for functional enrichment analysis and KEGG analysis. The results showed that the biological processes (BP) were mainly involved in “cytokine-mediated signaling pathway”, “cellular response to cytokine stimulus”, “response to cytokine”, and “cell migration” (Fig 3C). The most enriched ‘cellular components (CC)’ were “extracellular region”, “extracellular space”, “cell surface”, and “side of membrane” (Fig 3D). Regarding the molecular function (MF) analysis, the most relevant items of the hub genes were “signaling receptor binding”, “signaling receptor activity”, “molecular transducer activity”, and “cytokine receptor binding” (Fig 3E). The results of KEGG pathway analyses clearly suggested that the 15 hub genes were significantly enriched in the “cell adhesion molecules (CAMs)”, “pathways in cancer”, “malaria”, and “leukocyte transendothelial migration (LTM)” (Fig 3F). Based on the results of functional enrichment and pathway analysis, we concluded that the 15 hub genes significantly involved in the LTM. Among the 15 identified hub genes, while several genes such as PECAM1, VCAM1, CXCL8, and CCL2 are well-established mediators of general leukocyte adhesion and chemotaxis, we specifically focused on MMP2 and ITGB2 for further analysis. This selection was based on the following rationale: Firstly, MMP2 (Matrix Metalloproteinase 2) plays a dual role not only in facilitating leukocyte migration but also in the degradation and remodeling of the extracellular matrix (ECM), a hallmark pathological process in liver cirrhosis. Secondly, ITGB2 (Integrin Subunit Beta 2) is a critical adhesion molecule specifically expressed on leukocytes, mediating the firm adhesion step crucial for transendothelial migration. We hypothesized that these two genes might represent a specific link between the inflammatory response and fibrotic progression in LC.Therefore, we selected 2 LTM-related genes (MMP2, ITGB2) from the 15 hub genes for further analysis.

### 3.5. Analysis of the LC immune microenvironment

**Fig 4:**
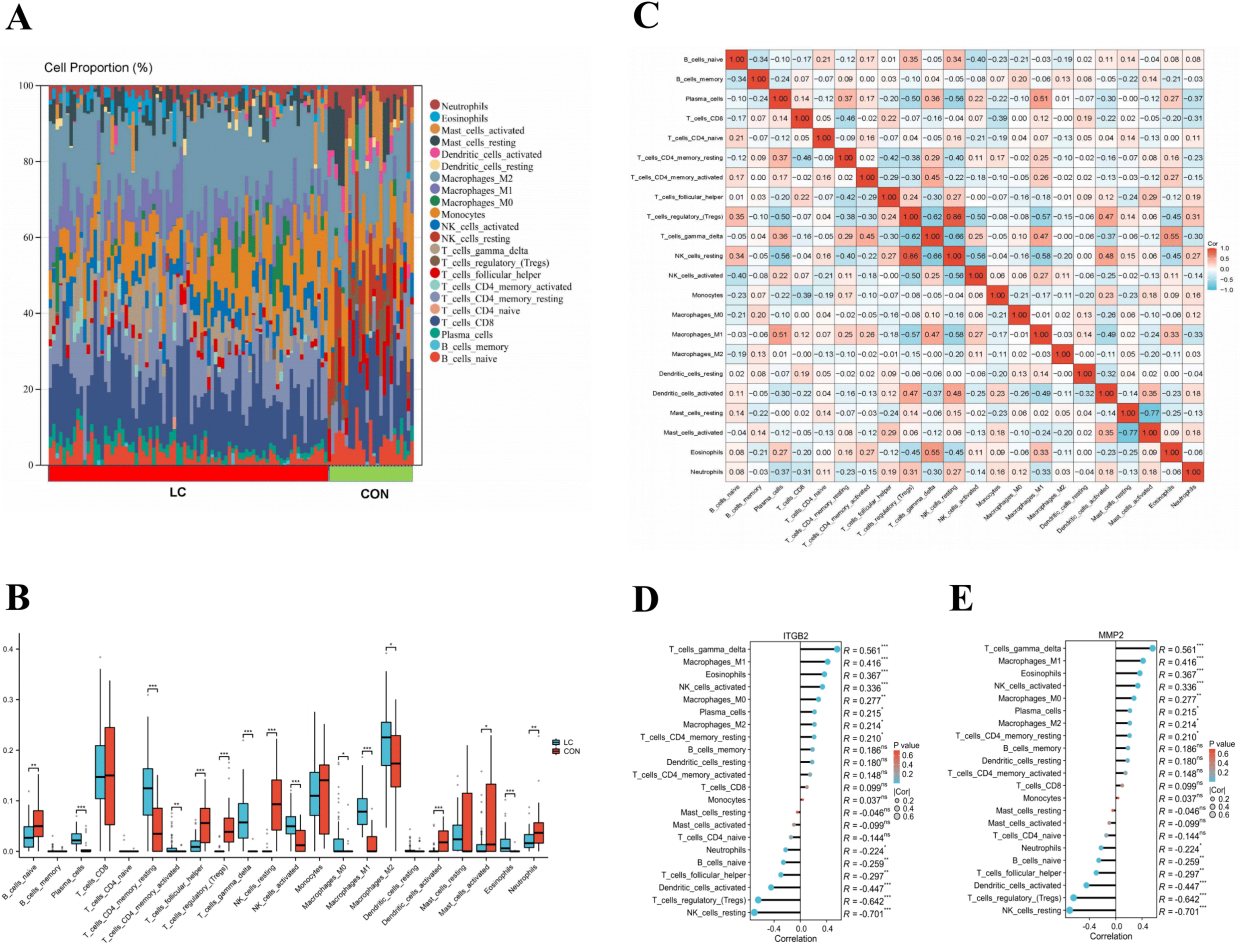
Immune microenvironment analysis in liver cirrhosis(LC). **(A)** Stacked histogram displaying the immune cell proportions between LC and control groups. **(B)** Violin plot showing the comparison of 22 kinds of immune cells between LC and control groups. **(C)** The heatmap revealing the correlation of 22 kinds of immune cells infiltration. The correlation map representing the associations of the infiltrated immune cells with **(D)** integrin β2(ITGB2) and **(E)** matrix metallopeptidase 2(MMP2).

Based on the CIBERSORT algorithm, we estimated the relative proportion of 22 types of infiltrated immune cells (Fig 4A, B) and the correlations between the infiltrations of immune cells in the unified LC dataset (Fig 4C). The results revealed that seven types of immune cells had a positive relationship with LC development, including plasma cells, resting memory CD4 T Cells, activated memory CD4 T Cells, gamma delta T cells, activated NK cells, macrophages M0, macrophages M1, macrophages M2, and Eosinophils. On the contrary, seven types of immune cells were responsible for the negative correlation to LC, including naive B cells, follicular helper T cells, regulatory T cells, resting NK cells, activated dendritic cells, activated mast cells, and neutrophils. We also found correlations between immune cell phenotypes, suggesting that the interaction between immune cells may play an important role in LC development. For identifying the immune correlation of 2 LTM-related genes (MMP2, ITGB2), we employed the method of Spearman correlation analysis to analyse the correlation between the 2 hub genes and 22 immune cells in LC (Fig 4D, E). The results indicated that the expression level of MMP2 was positively associated with gamma delta T cells, macrophages M1, eosinophils, activated NK cells, macrophages M0, plasma cells, macrophages M2, and resting memory CD4 T Cells. In addition, MMP2 expression was negatively correlated with resting NK cells, regulatory T cells, activated dendritic cells, follicular helper T cells, naive B cells, and neutrophils. Likewise, ITGB2 expression positively correlated with gamma delta T cells, Eosinophils, macrophages M0, macrophages M1, activated NK cells, and macrophages M2. Moreover, ITGB2 expression levels were negatively correlated with resting NK cells, regulatory T cells, activated dendritic cells, and naive B cells. Thus, MMP2 and ITGB2 may contribute to LC progression through regulating the immune microenvironment.

### 3.6. The evaluation of the diagnostic and prognostic clinical value of the two LTM-related genes in LC

**Fig 5:**
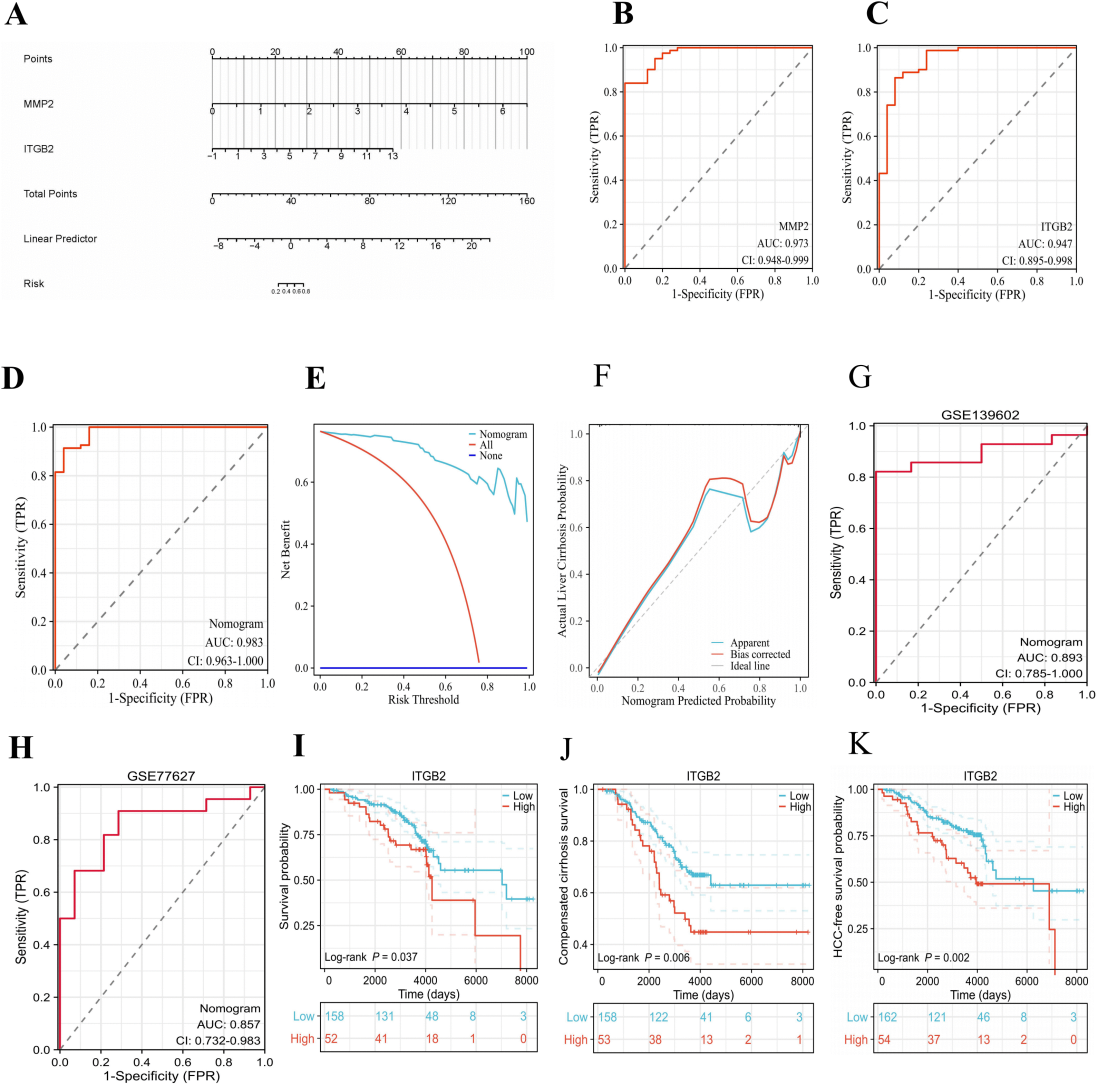
Evaluation of the diagnostic performance of the nomogram model in distinguishing liver cirrhosis(LC) and the prognostic predictive power of integrin β2(ITGB2) in LC patients. **(A)** The nomogram was conducted based on the expression of ITGB2 and matrix metallopeptidase 2(MMP2) to predict the risk of occurring LC. **(B, C, D)** The Receiver Operating Characteristic‌(ROC) curves of each candidate biomarker (ITGB2 and MMP2) and nomogram showed the diagnostic power in predicting the risk of occurring LC. **(E)** Decision Curve Analysis(DCA) for the nomogram. **(F)** The calibration curve of nomogram prediction in LC patients. **(G, H)** The ROC curve derived using the nomogram showed the diagnostic capabilities for LC in the validation datasets (GSE139602 and GSE77627). **(I, J, K)** Kaplan-Meier plots of survival probability, compensated cirrhosis probability and ‌Hepatocellular Carcinoma(HCC)-free survival probability of the LC patients‌‌ stratified by low vs. high expression of ITGB2 in the validation data set (GSE15654).

To evaluate the ability of the two LTM-related genes (MMP2, ITGB2) to serve as diagnostic biomarkers in LC, we constructed a diagnostic nomogram model based on the unified LC dataset to predict the likelihood of LC diagnosis (Fig 5A). The ROC analysis results showed that the nomogram model had the highest AUC (0.983) when compared with that of each biomarker (Fig 5B, C, D). The DCA curves and calibration curves also proved the good predictive ability of the nomogram model (Fig 5E, F). The nomogram also exhibited high diagnostic accuracy for LC in two independent external datasets, achieving AUCs of 0.893 in GSE139602 and 0.857 in GSE77627 (Fig 5G, H). To evaluate the clinical relevance of the two LTM-related gene expression in LC, we extracted the gene expression levels of the two LTM-related genes and patient prognosis data from the full microarray dataset of a similar study (GSE15654). Kaplan-Meier survival analysis indicated that high ITGB2 expression was significantly associated with poorer overall survival (OS), shorter compensated cirrhosis survival, and reduced hepatocellular carcinoma (HCC)-free survival compared to low expression groups (Fig 5I, J, K). These findings suggest that ITGB2 may serve as a potential prognostic biomarker for LC progression. However, due to the lack of detailed clinical covariates in the current public dataset, we were unable to perform multivariate analysis to confirm its status as an independent prognostic factor.

### 3.7. The small molecular compound docking of the two LTM-related genes

**Fig 6:**
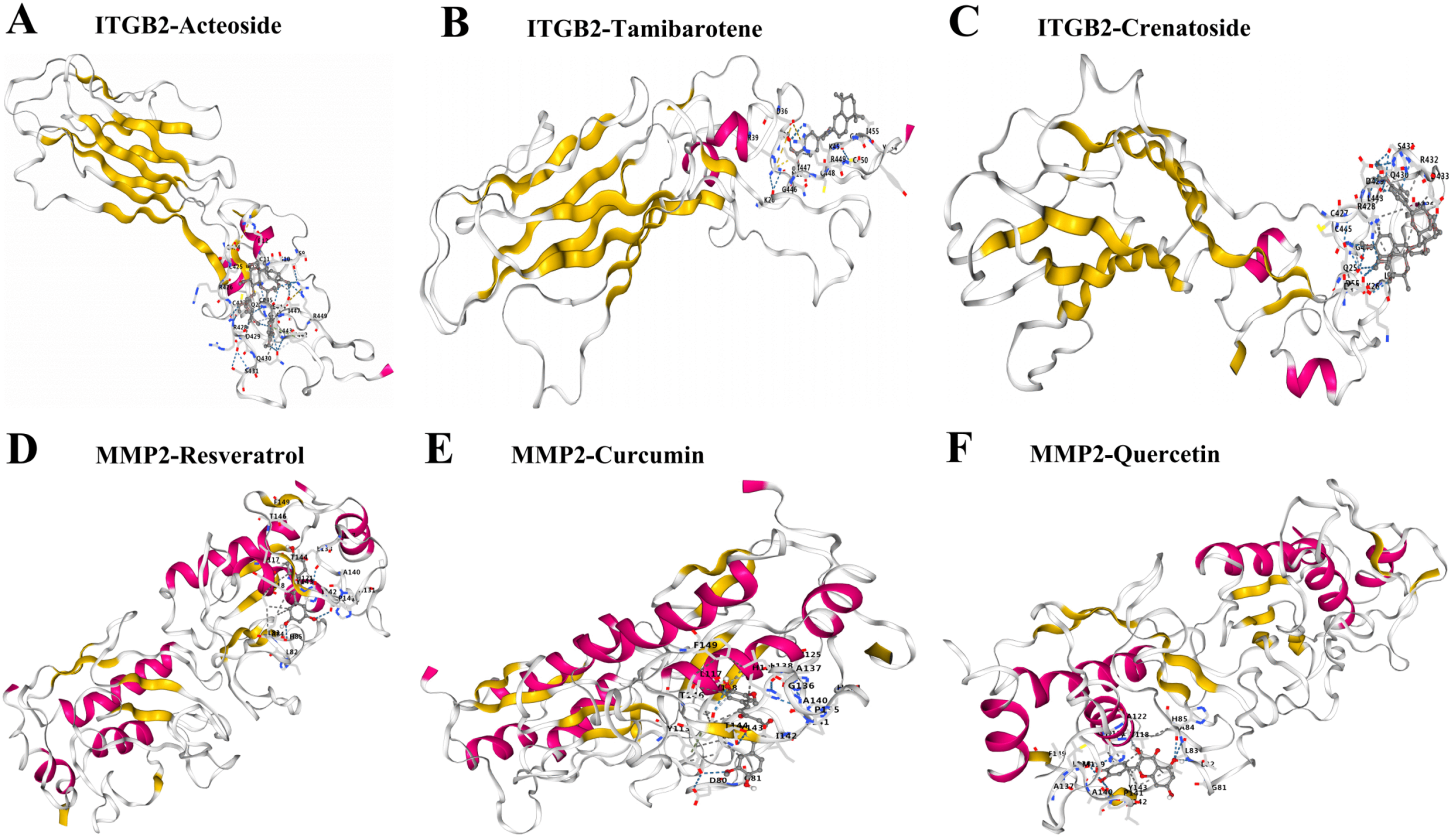
The docking results of the proteins encoded by integrin β2(ITGB2) and matrix metallopeptidase 2(MMP2) with small molecular compounds. The predicted binding modes of ITGB2 with acteoside (A), tamibarotene (B), and crenatoside (C). The predicted binding modes of MMP2 with resveratrol (D), curcumin (E), and quercetin (F).

By molecular docking, we explored the drugs targeted to the two LTM-related genes (MMP2, ITGB2). Based on the docking scoring results, we identified promising candidate compounds that exhibit strong binding affinity to the active sites of ITGB2, including acteoside, tamibarotene, and crenatoside (Fig 6A, B, C). The candidate drugs were also evaluated through molecular docking to determine the potential for physical interactions between the drugs and the MMP2 protein, which serves as a drug target. Similarly, several drugs, including resveratrol, curcumin, and quercetin, showed tight binding poses with MMP2, suggesting their potential as inhibitors of MMP2 (Fig 6D, E, F).

### 3.8. Immunohistochemistry of MMP2 and ITGB2 expression in liver tissues

**Fig 7:**
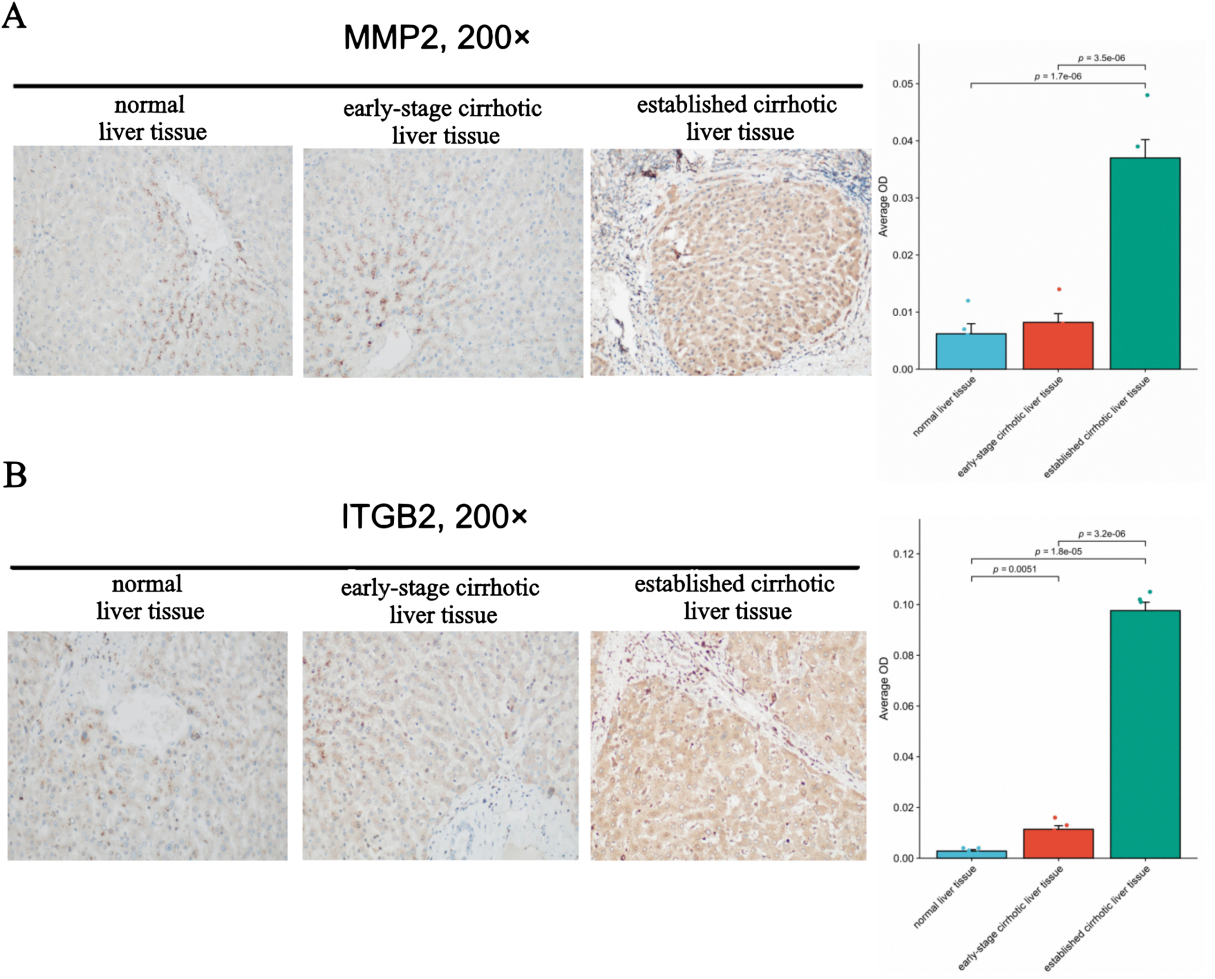
Immunohistochemical results of matrix metallopeptidase 2(MMP2) and integrin β2(ITGB2) in liver tissues. **(A)** Immunohistochemical results of MMP2 in normal, early-stage cirrhotic and established cirrhotic liver tissues. **(B)** Immunohistochemical results of ITGB2 in normal, early-stage cirrhotic and established cirrhotic liver tissues.

In this study, liver tissue samples from a total of 15 patients undergoing hepatic lobectomy for hepatolithiasis were collected. Among these samples, 5 were diagnosed as normal liver tissue, 5 as having early-stage LC, and 5 as having established LC. The diagnosis of LC was made using the Ishak fibrosis score (F) ranged from 0–6 (F0 = none, F1 = early portal, F2 = established portal, F3 = early bridging, F4 = established bridging, F5 = early cirrhosis, and F6 = established cirrhosis). Immunohistochemical analysis revealed that MMP2 expression was significantly elevated in established cirrhotic liver tissues compared to both normal liver tissues and the early-stage cirrhotic liver tissues. In contrast, no significant difference in MMP2 expression was observed between the early-stage cirrhotic liver tissues and normal liver tissues (Fig 7A). Furthermore, ITGB2 expression was significantly elevated in established cirrhotic liver tissues relative to normal and early-stage cirrhotic liver tissues. Moreover, a significant increase in ITGB2 expression was also observed in early-stage cirrhotic liver tissues compared to normal liver tissues (Fig 7B). Thus it can be seen, MMP2 and ITGB2 may be the specific biomarkers for established LC.

## 4. Discussion

Liver cirrhosis (LC), an advanced stage of chronic liver disease, not only compromises patient prognosis by predisposing individuals to liver failure and hepatocellular carcinoma but also imposes a substantial medical and socioeconomic burden. [18,19]. Current diagnostic methods have limitations in early-stage detection, and treatment options mainly focus on symptom management and preventing complications [20,21]. Thus, this study is crucial for exploring new diagnostic and therapeutic strategies. We have demonstrated the important roles of the two LTM-related genes (MMP2, ITGB2) in LC progression. Although previous studies have implicated MMP2 and ITGB2 in liver diseases, a comprehensive, multi-dimensional bioinformatic analysis focusing specifically on their roles within the LTM pathway in LC has been lacking. Unlike general inflammatory markers, our study highlights that MMP2 serves as a bridge between immune cell infiltration and hepatic fibrosis due to its ECM remodeling capabilities. Furthermore, the construction of the diagnostic nomogram based on these two genes, combined with the identification of potential therapeutic agents through molecular docking, provides a novel perspective that was not fully explored in prior research. MMP2 and ITGB2 therefore may be potential therapeutic targets for treatment of LC.

Previous studies have confirmed that the persistence of inflammatory responses drives the activation of hepatic stellate cells, leading to liver fibrosis and ultimately liver failure [22,23]. LTM represents an important link in launching an inflammatory immune response [24]. Thereby, LTM may be closely related to LC development and progression. However, there is lack of additional dedicated studies exploring the exact relationship between LTM and LC. In this study, we provide the preliminary evidence supporting the important regulatory function of LTM in LC development and progression.

In order to discover the key regulatory genes of LC development, we first screened for the differentially expressed genes between LC and normal liver tissues. Subsequently PPI network analysis and 4 algorithms from cytoHubba had been used to identify 15 key regulatory genes. We then performed functional enrichment analysis to explore the specific mechanisms by which the key genes would regulate LC development.

In our study, enrichment analysis of key genes showed that the possible mechanisms for LC development converged on “transmission of signaling molecules”, “cell adhesion molecules”, “pathways in cancer”, and “leukocyte transendothelial migration (LTM)”. Because multiple cell types and cytokines are thought to be involved in LC initiation and progression, the pathological mechanism of LC involves a variety of signaling pathways, including cancer disease pathways [25–27]. Nonetheless, there is less reported evidence of LTM effects on LC development. As a primary component of the inflammatory responses, LTM plays an important role in inducing hepatic inflammatory responses to injury. Previous studies have shown that inflammatory responses promote the differentiation of hepatic stellate cells (HSCs) into matrix-producing myofibroblasts, eventually culminating in liver fibrosis and cirrhosis [28]. It is therefore reassuring that our results about the roles of LTM in LC development are consistent with previous studies.

From our findings, we selected 2 LTM-related genes (MMP2, ITGB2) as the potential key regulators of LC development for in depth analysis. Since LC development is intimately tied to the immune cell infiltration, we first set out to explore the roles of MMP2 and ITGB2 in regulating immune infiltration. We found that MMP2 and ITGB2 were significantly correlated with immune cell hepatic infiltration in LC. MMP2 is one of zinc-dependent matrix metalloproteinases and its main role is likely to degrade and remodel the extracellular matrix (ECM). Dysregulation of MMP2 can mediate excessive ECM degradation, leading to tissue scarring [29]. Abnormal expression of MMP2 is often associated with multiple physiological and pathological mechanisms, including immune cell infiltration in liver tissues [30]. For example, MMP2 can promote the recruitment of fibroblasts, creating a physical barrier to immune infiltration [31]. Integrin β2（ITGB2）, a integrin subunit, is widely expressed in leukocytes, which mediates leukocyte adhesion and migration, and it has important effects on inflammation and immune responses [32]. Our results suggest that MMP2 and ITGB2 can influence LC development through a mechanism that involves regulation of immune infiltration.

Our study further found ITGB2 held potential diagnostic and prognostic value for LC, while MMP2 had only diagnostic value for LC.Currently, there is a lack of specific and sensitive clinical indicators for early diagnosis and prognosis judgment in LC patients. MMP2 and ITGB2 are expected to be the potential diagnostic and prognostic indicators of LC. Not only that, MMP2 and ITGB2 can be detected non-invasively in most bodily fluids such as serum, which is favourable for practical applications. However, larger sample sizes are needed to evaluate the clinical value of MMP2 and ITGB2 in LC patients. Based on the above results, MMP2 and ITGB2 could be the important targets for future LC treatment strategies. Therefore, we used molecular docking to identify several promising candidates, such as the natural compounds Curcumin and Resveratrol, as potential inhibitors targeting MMP2 and ITGB2. Previous studies were primarily focused on discovery of the roles of resveratrol, curcumin, and quercetin in LC development. For example, resveratrol could alleviate liver fibrosis through NF-KappaB, TLR2, TNF-alpha, TGFbeta1, and other inflammatory signaling pathways [33–35]. Investigations of the regulatory mechanisms of curcumin in the development of LC are mainly focused on the regulation of oxidative stress and autophagy [36,37]. The mechanisms by which quercetin reverses experimental cirrhosis are mainly the regulation of the proinflammatory process and oxidative stress [38,39].Whether resveratrol, curcumin, and quercetin can alleviate liver fibrosis by regulating MMP2 and ITGB2 requires future study. The possible benefits of acteoside, tamibarotene, crenatoside for the treatment of LC also need to be further explored. Moreover, although the docking results are promising, direct experimental evidence confirming that these compounds effectively inhibit MMP2/ITGB2 function or alleviate LC in vivo is still lacking. In this study, Immunohistochemistry (IHC) was used for supplemental analysis to make up for the deficiency of pure bioinformatics analysis. The observed overexpression of MMP2 and ITGB2 in cirrhotic liver tissues indicates a correlation between LTM-related genes and the progression of LC, implying their potential involvement in the disease process.

This study still has several research limitations. Foremost, our study on the mechanism by which LTM-related genes regulate LC development has not been delved deep enough. We acknowledge that the precise functional roles, specific signaling pathways, and cellular origins of MMP2 and ITGB2 require further experimental investigation. For instance, future studies could utilize HepG2 cells to validate the regulatory effects of MMP2 on inflammation and immune cell migration [40]. Second, this study has relatively small sample sizes due to the limited public alternative LC data currently. Although external validation mitigates overfitting, limited sample sizes and potential batch effects persist. Large-scale, prospective studies are warranted to confirm clinical utility. Third, while Kaplan-Meier analysis confirmed the prognostic value of ITGB2, incomplete clinical data precluded the assessment of its independence via multivariate Cox regression. Future studies with comprehensive data are warranted. Finally, while molecular docking suggested several small-molecule compounds as potential therapeutic agents, these findings are purely computational predictions. We strictly acknowledge that docking cannot prove that these compounds reduce gene/protein expression or have therapeutic efficacy; rigorous in vitro enzyme activity assays and in vivo functional ‌‌studies are required to validate these interactions in future work.

In summary, the progression of LC may be influenced by LTM-related genes, particularly MMP2 and ITGB2. These genes appear to be involved in modulating hepatic inflammation and immune responses during LC progression, indicating their potential as diagnostic and therapeutic targets.
